# Using a Digital-Based Mindfulness Curriculum to Enhance Healthy Aging Outcomes in Community-Dwelling Older Adults in Taiwan: Mixed Methods Feasibility Study

**DOI:** 10.2196/84161

**Published:** 2026-05-13

**Authors:** Yu-Rung Wang, Pei-Lun Hsieh, Chia-Hsiu Chang, Chia-Chi Hsiao, Mei-Lien Hu

**Affiliations:** 1 Department of Nursing and Center for Smart Healthcare Education, Chang Gung University of Science and Technology, Chiayi Campus, Puzi, Taiwan; 2 Department of Nursing, National Taichung University of Science and Technology, Taichung, Taiwan; 3 Department of Nursing, Hungkuang University, Taichung, Taiwan; 4 Chang Gung Medical Foundation, Chiayi Chang Gung Memorial Hospital, Puzi, Taiwan; 5 Department of Nursing, Linkou Chang Gung Memorial Hospital, 5 Fuxing St, Guishan District, Taoyuan City, 333, Taiwan, 886 3-328-1200 ext 5521

**Keywords:** mindfulness, older adults, healthy aging, Delphi method, mixed methods, digital learning

## Abstract

**Background:**

Taiwan’s status as a super-aged society underscores the need for efficient strategies to promote healthy aging. While the benefits of mindfulness-based interventions for sleep and mental health are evident, there is a shortage of cultural adaptations for Taiwan’s older adults. Current courses mainly focus on mindfulness-based stress reduction, while neglecting mindfulness-based elder care, and mindful sustainable aging principles. However, the abstract nature of some mindfulness concepts, combined with limited digital support and localized resources, makes it challenging for older adults to engage effectively.

**Objective:**

To enhance healthy aging outcomes in older community-dwelling adults in Taiwan, this study developed an 8-week theory-based mindfulness curriculum that combined the mindfulness-based stress reduction, mindfulness-based elder care, and mindful sustainable aging frameworks with digital health support.

**Methods:**

This research employed a mixed methods feasibility study design. We used the modified Delphi method in phase 1 to reach agreement on curriculum content and digital material selection. Ten older adults participated in the pilot study, which comprised phase 2. We used quantitative measurements to assess mindfulness, sleep quality, cognition, heart rate variability, perceptions of aging, healthy aging perspectives, and physical function and activity at baseline, at weeks 4 and 8, and at a 1-month follow-up. Qualitative interviews were conducted to gather insights into participants’ experiences.

**Results:**

Participants achieved significant improvements in mindfulness, sleep quality, aging perception, healthy aging outlook, and physical function during the study (all *P*<.05). Cognition, heart rate variability, and physical activity levels showed no significant changes throughout the study. The qualitative data supported these findings, as participants selected simple practices, such as mindful breathing, which they practiced daily to enhance their emotional well-being and social connections. Participants valued the digital learning materials for aiding their practice; however, some older adults with reading difficulties faced challenges accessing the content.

**Conclusions:**

Using the Delphi method resulted in an acceptable and feasible theory-based digitally supported mindfulness program that improved several indicators of healthy aging in older adults in Taiwan. Our findings need to be validated using longer trials to assess cognitive and physiological effects. Furthermore, digital accessibility requires further development.

## Introduction

Taiwan is rapidly becoming a super-aged society, prompting the government to develop long-term care policies aligned with the World Health Organization’s (WHO) Decade of Healthy Aging framework [1,2]. Current community programs in Taiwan emphasize physical exercise and cognitive training but lack adequate organized support for mental health and attitudes toward aging [3-5]. The WHO model defines healthy aging as the maintenance of functional abilities alongside the management of ongoing health conditions and external environmental challenges. Recent research suggests that healthy aging involves 3 key elements—an individual’s wellness status, their ability to adapt, and their capacity to resist challenges—all while accounting for the mental and social aspects of aging [2,4,5]. Supporting older adults requires interventions that address both physical and psychological factors to help preserve their intrinsic capacity and functional ability.

According to Kabat-Zinn [6], mindfulness practice enables individuals to experience the present moment without judgment. Research has indicated that mindfulness practice can improve physical and mental health across different groups [7-11]. Moreover, mindfulness-based stress reduction (MBSR) has been shown to enable participants to reduce stress symptoms, improve sleep quality, and achieve better mental health outcomes [9-11]. Although older adults who participate in mindfulness-based interventions (MBIs) have demonstrated better sleep quality and higher life satisfaction, such programs have not shown positive effects on cognitive functions [12-14]. Mindfulness practices thus have the potential to help seniors during their aging journey, but they require consideration of the particular needs of older adults.

As older adults are still able to learn and develop skills, multiple frameworks have been developed for mindfulness practice during senior years. For example, the mindfulness-based elder care (MBEC) program aims to teach frail older adults and their caregivers to practice mindfulness [15-17]. The mindful sustainable aging (MSA) framework unites successful aging principles with disengagement, transcendence, and activity theory to help older adults maintain their outward activities while developing inner reflection and resilience [18,19]. These approaches, together with the core stress-reduction practices of MBSR [6,9,10], establish a strong theoretical foundation for mindfulness programs that focus on emotional regulation, the acceptance of aging, and the development of purpose for older adults. Community-based programs targeting this population therefore require interventions that combine these particular frameworks to achieve their goals.

The majority of mindfulness programs for older adults in Taiwan use MBSR as their primary format; however, these programs do not directly implement MBEC and MSA principles and thus may not address specific needs of older populations. Older participants may have difficulty understanding abstract concepts such as the “nine attitudes of mindfulness” (ie, nonjudging, patience, beginner’s mind, trust, nonstriving, acceptance, letting go, gratitude, and generosity) [20]. Since older adults may lack classroom experience and have limited memory capacity, teachers can facilitate their learning through practical, hands-on activities rather than abstract examples. Additionally, the lack of suitable, culturally adapted, age-friendly materials for older Taiwanese adults can contribute to poor adherence after course completion.

Taiwan’s digital health agenda encourages the development of digital learning programs that can be integrated into community-based services for older adults [21]. About half of Taiwanese adults aged65 years and above use the internet, and many are familiar with LINE (LY Corporation), a widely used communication and social media application [21]. Digital platforms incorporating reminder systems may support older adults in monitoring medical treatment schedules and making health-related behavioral changes [13]. However, developers should ensure that digital solutions are accessible to users who have reading difficulties, mistrust of web-based information, or difficulty operating digital systems.

Existing research has demonstrated a lack of mindfulness programs that are culturally adapted for Taiwanese older adults and that integrate basic digital tools while adhering to established principles of mindfulness and healthy aging. To address this gap, this study adopted a mixed methods approach and conducted a feasibility study. Our research team developed an 8-week mindfulness program for community-dwelling older adults in Taiwan, integrating components of MBSR, MBEC, and MSA. Home-practice support was provided through LINE and YouTube. The study had two primary aims: (1) to apply a modified Delphi approach to achieve expert consensus regarding curriculum content and digital educational resources and (2) to assess the program’s feasibility, participant acceptance, and initial impact on mindfulness levels, sleep quality, cognitive ability, heart rate variability (HRV), perceptions of aging, physical activity, and overall physical functioning. Based on established evidence-based approaches, an initial digital mindfulness program framework incorporating proven methods was created and adapted to support Taiwanese older adults in managing the psychosocial and functional aspects of healthy aging through digital health support.

## Methods

### Study Design

**Figure 1.**
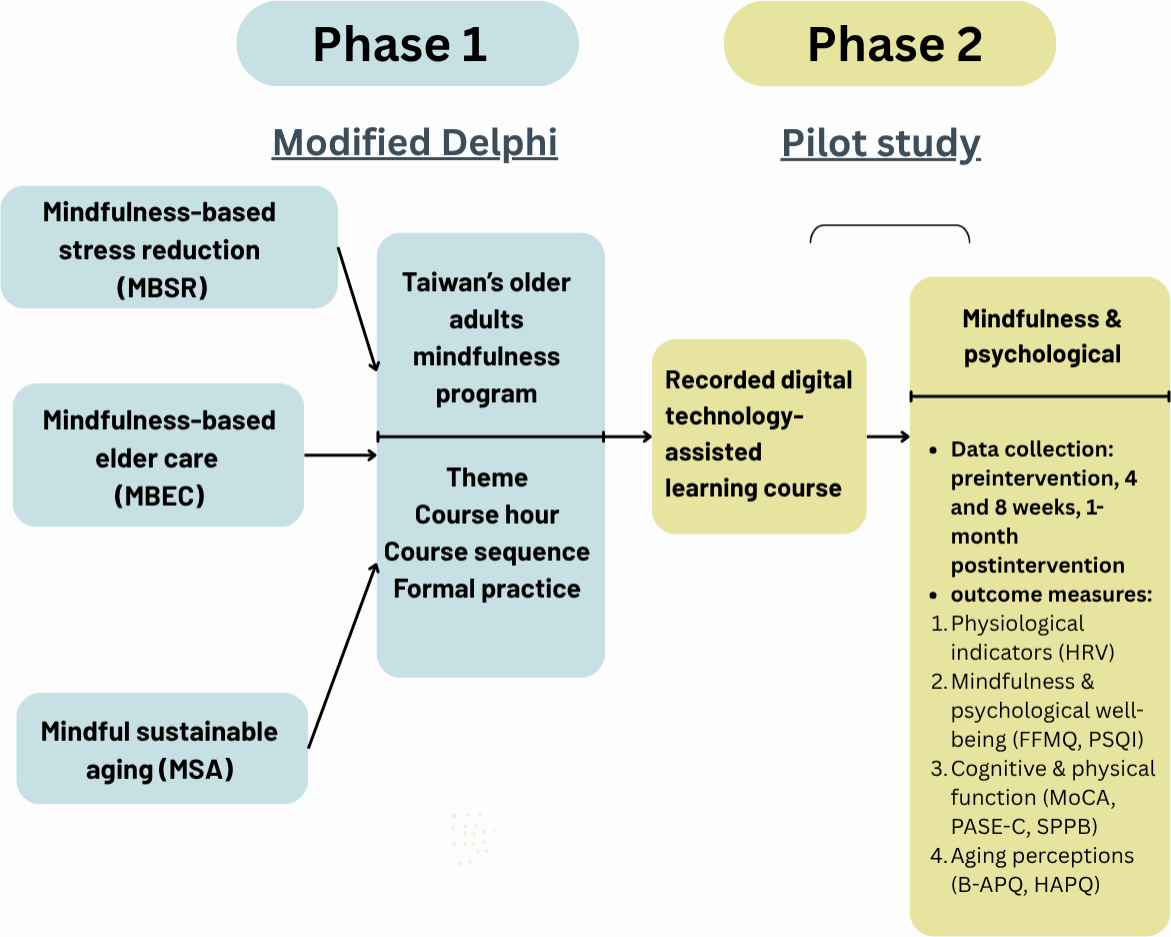
Conceptual and methodological framework of the 2-phase study: Delphi curriculum development and pilot intervention. B-APQ: Brief Aging Perceptions Questionnaire; FFMQ: Five Facet Mindfulness Questionnaire; HAPQ: Healthy Aging Perspectives Questionnaire; HRV: heart rate variability; MoCA: Montreal Cognitive Assessment; PASE-C: Chinese Physical Activity Scale for the Elderly; PSQI: Pittsburgh Sleep Quality Index; SPPB: Short Physical Performance Battery.

The mixed methods feasibility design was based on Creswell and Plano Clark [22] (Figure 1). Phase 1 used a modified Delphi process to develop and validate a theory-based, digitally supported mindfulness curriculum tailored for community-dwelling older adults. Phase 2 consisted of a single-group pilot study with embedded qualitative interviews (QUAN→qual) to evaluate feasibility and acceptability and explore the curriculum’s preliminary effects on healthy aging outcomes.

### Phase 1: Delphi Curriculum Development

#### Theoretical Framework and Curriculum Design

We used a modified Delphi approach to determine the appropriate format and content of a mindfulness-based program for community-dwelling older adults. The curriculum incorporated 3 theoretical frameworks: MBSR [6], which teaches mindfulness practices and stress reduction for daily life; MBEC [15], which focuses on mindfulness practice in gerontological care settings; and MSA [19], which promotes mindfulness to help individuals improve adaptability and resilience in later life. The curriculum development process also included the creation of digital learning resources. These resources supported participants between in-person program sessions, facilitated adherence to home practices, and enhanced the intervention’s ecological validity.

#### Participants

Experts were purposefully recruited from professional networks and academic institutions. Eligible participants included certified mindfulness instructors, physicians, nurses, physical and occupational therapists, schoolteachers, health educators, and case managers with a minimum of 2 years of experience in mindfulness instruction or elder care (Table S1 in Multimedia Appendix 1).

#### Procedure

The Delphi process was administered via mailed questionnaires across 3 sequential rounds conducted over approximately 6 months, with each round lasting approximately 2 months, including time for qualitative feedback synthesis and item revision. During the first round, the experts reviewed the curriculum materials and provided qualitative suggestions, which were synthesized and transformed into structured items. In the subsequent 2 rounds, experts rated these items quantitatively and offered additional feedback. This iterative process continued until consensus was achieved. The finalized 8-week curriculum and digital learning materials were then implemented and evaluated in phase 2 at a community care station in Chiayi City.

#### Data Analysis

Descriptive statistics were calculated for the Delphi survey responses. For each curriculum item, the median, IQR, and percentage of experts rating the item as “suitable” or “very suitable” were calculated. Consensus was defined a priori as an IQR≤1.0 and at least 80% agreement among experts [22]. Items that did not reach consensus were revised according to experts’ comments and resubmitted in the next round. This process continued until response stability was achieved.

### Phase 2: Pilot Intervention Study (QUAN→Qual)

#### Quantitative Study

The second phase comprised a single-group pilot intervention involving 10 community-dwelling older adults. Participants completed standardized assessments at 4 time points: baseline (P0), week 4 (P1), week 8 (P2), and 1-month postintervention (P3). The repeated-measures longitudinal design allowed examination of both the immediate and long-term effects of the intervention on healthy aging outcomes.

#### Digital Support Component

The intervention combined in-person instruction with digital and communication tools to support participants in their mindfulness practice between sessions.

**Figure 2.**
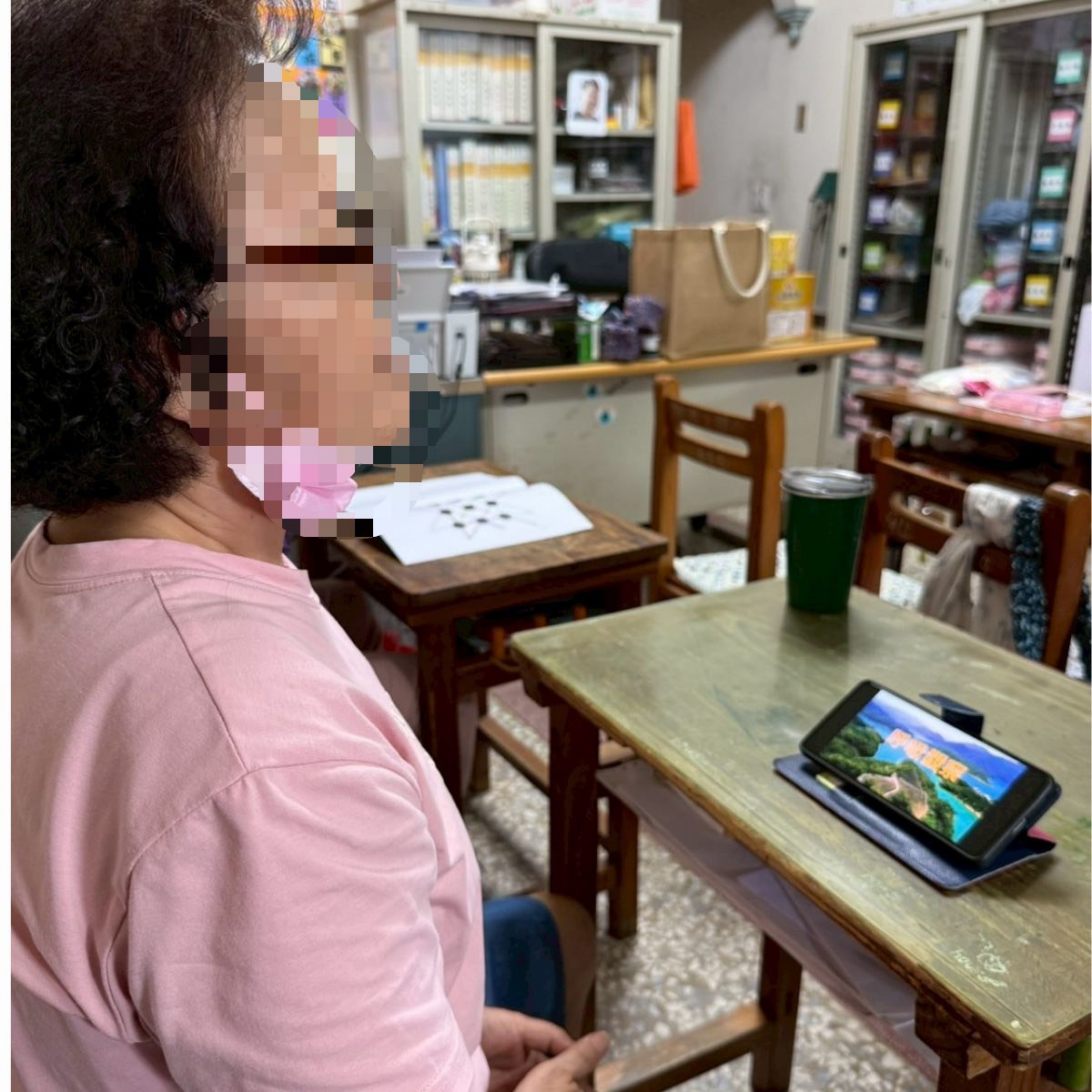
Older adult using digital learning materials on the YouTube platform.

**Figure 3.**
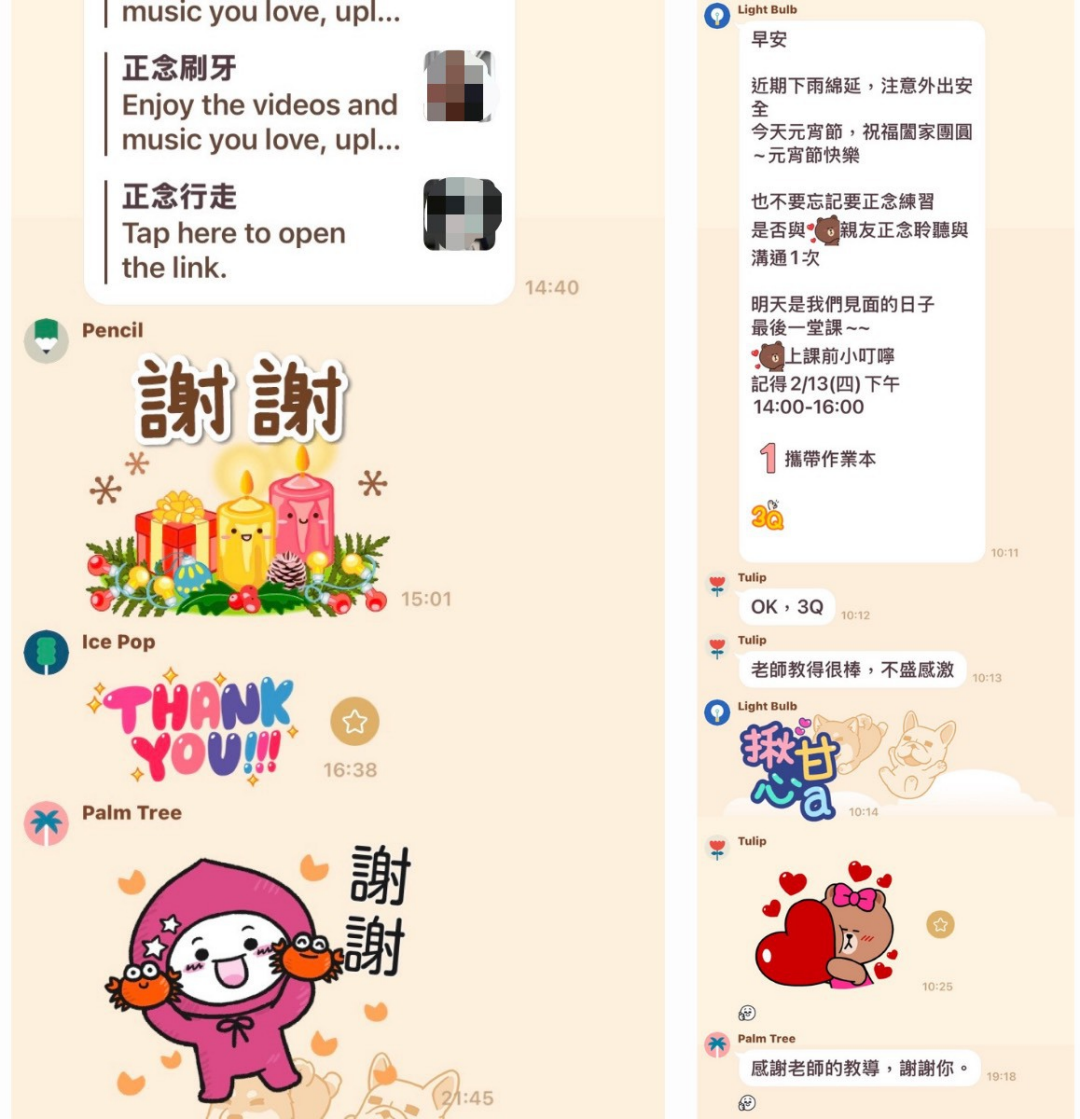
LINE app group for mindful practice and real-time interaction.

Two platforms were used:

- YouTube content: short instructor-recorded videos and audio-guided sessions demonstrating core practices—such as mindful breathing, body scans, gentle stretching, and mindful walking—were made available through YouTube. Participants could access these materials at home to support independent practice and reinforce what they had learned in class (Figure 2).
- LINE reminders and group chat: A LINE group was created for the class to facilitate communication. Brief practice reminders and encouraging messages were sent to participants up to 3 times daily (morning, noon, and evening), along with links to relevant YouTube content and simple emotional support cards. The group also provided a means for participants to ask questions, share experiences, provide mutual support, and maintain engagement between sessions (Figure 3).

This hybrid format combined the social advantages of in-person sessions with accessible, age-appropriate digital tools already familiar to participants. The perceived usefulness of these digital components was assessed through postprogram qualitative feedback; objective engagement metrics, such as video view counts or message read rates, were not collected.

#### Qualitative Component: In-Depth Interviews

One week after completing the 8-week program, all participants were invited to take part in face-to-face, in-depth interviews to discuss their experiences with the curriculum and digital support materials. Interviews were conducted in a private room at the community care station by the course instructor, who was trained in mindfulness facilitation and qualitative interviewing. Sessions lasted approximately 30 minutes and followed a semistructured interview guide (Multimedia Appendix 2), encompassing questions regarding participants’ personal experiences, any benefits they experienced, and their recommendations for improvements.

To minimize social desirability bias arising from the instructor-participant relationship, participants were assured that their responses would not affect their continued participation or access to services. They were encouraged to share both positive and negative experiences and reminded that they could decline to answer any question. With participants’ consent, the interviews were audio-recorded and transcribed verbatim in Mandarin; field notes were taken during or immediately after each session. Transcripts were reviewed for accuracy prior to analysis.

### Participants

Participants were recruited from a community care station in Chiayi City offering frailty prevention and disability-delay programs. Recruitment was initiated through announcements at Long-Term Care Service C Stations, followed by referrals from social workers and case managers. These stations are community centers that provide primary long-term care by integrating disability prevention strategies, connecting preventive care with active aging programs to reduce physical decline, promoting health and well-being, and improving quality of life [1].

The eligibility criteria were as follows: (1) aged 65 years or older, (2) able to communicate in Mandarin or Taiwanese and participate in group discussions, (3) owning and regularly using a smartphone and being familiar with the LINE application, and (4) willing and able to attend all scheduled sessions during the intervention period. The exclusion criteria included (1) a prior diagnosis of cognitive impairment (eg, dementia), (2) severe hearing, speech, or communication difficulties that would prevent participation in group activities, and (3) severe or unstable physical conditions that would make participation unsafe or impractical. Ten older adults were ultimately enrolled in the pilot study.

### Ethical Considerations

Each participant signed a written consent form prior to study participation. We received ethical approval for this study from the Chang Gung Medical Foundation institutional review board (number 202400087B0). Before data collection, the research team explained the study objectives, procedures, participants’ rights, and measures to protect confidentiality and research data to all eligible participants. Participation was voluntary, and participants were assured they could discontinue at any stage of the study without penalty or negative impact. Original paper documents were kept in locked cabinets at the study institution. After the removal of personally identifiable information, research data were maintained in institutional cloud-based storage systems and on password-protected computers, and access was restricted to approved members of the research team. Data will be preserved until 2027 and subsequently destroyed in accordance with institutional data management requirements. A signed copy of the informed consent form was provided to each participant. To acknowledge their time and contribution, participants received a NT$100 gift voucher, approximately US $3, for each completed study-related task.

### Measures

#### Quantitative Outcome Measures

Quantitative data were collected at all 4 time points (P0-P3) using the following validated instruments:

- Mindfulness: Taiwanese version of the Five Facet Mindfulness Questionnaire (T-FFMQ; 39 items, higher scores indicate greater mindfulness) [7,23].
- Cognitive function: Taiwanese version of the Montreal Cognitive Assessment (MoCA; score: 0‐30, higher scores indicate better cognition) [24,25].
- Sleep quality: Chinese Pittsburgh Sleep Quality Index (PSQI; global score: 0‐21, higher scores indicate poorer sleep) [26,27].
- Perceptions of aging: Chinese Brief Aging Perceptions Questionnaire (B-APQ) [28-30].
- Healthy aging perspectives: Healthy Aging Perspectives Questionnaire (HAPQ) [31].
- Physical activity: Chinese Physical Activity Scale for the Elderly (PASE-C) [32].
- Physical function: Short Physical Performance Battery (SPPB; score: 0‐12) [33].
- HRV: standard deviation of normal-to-normal (SDNN) and low frequency (LF)/high frequency (HF) ratio measured with a Taiwan Food and Drug Administration–approved BEATINFO heart rate sensor and application.

We selected these measures to capture multiple domains of healthy aging, including psychological, cognitive, and physical dimensions. Multimedia Appendix 3 provides detailed descriptions of each instrument and its psychometric properties.

#### Data Analysis

##### Quantitative and Qualitative Data Analysis

Quantitative and qualitative data were analyzed separately before integration. Descriptive statistics (mean, SD, frequency, and percentage) were used to characterize participants’ demographic data. Because of the small sample size and nonnormal distribution of the repeated-measures data, we employed the Friedman test for within-group comparisons across the 4 time points. For statistically significant results, post hoc pairwise comparisons were conducted to determine which time points showed meaningful changes. Statistical significance was set at *P*<.05 (2-tailed). All analyses were performed using SPSS Statistics version 25 (IBM Corporation). All 10 participants completed the 8-week intervention, all 4 quantitative assessments, and the postprogram interviews. Given no missing outcome data, analyses were conducted using complete cases (n=10) without the need for imputation.

##### Qualitative Analysis

We conducted thematic analysis following Braun and Clarke’s 6-phase framework (familiarization, coding, theme development, review, definition, and reporting) [34]. Two researchers individually coded the transcripts and resolved discrepancies through discussion. Codes were organized into subthemes and overarching themes reflecting participants’ experiences with the curriculum and digital practice materials. We applied investigator triangulation and peer debriefing methods, maintaining a coding decision audit trail to enhance trustworthiness. The selected quotes effectively represent the themes and use pseudonyms to protect participants’ identities.

##### Integration of Quantitative and Qualitative Findings

At the interpretation stage, quantitative and qualitative findings were merged to develop a comprehensive understanding of the feasibility, acceptability, and preliminary effects of the mindfulness curriculum.

## Results

### Phase 1: Delphi Consensus

The modified Delphi process yielded strong expert consensus on the 8-week mindfulness curriculum (Tables1  2). Qualitative feedback from round 1 was synthesized and used to revise items in subsequent rounds. In rounds 2 and 3, all items received strong consensus, with median scores of 4 and IQRs ≤1.0 across sessions. By round 3, agreement remained high, with at least 88% of experts rating each session as “suitable” or “very suitable” (Table 2). These results supported the curriculum’s content validity and expert acceptability, including its digital learning materials, and justified advancing to pilot feasibility testing.

**Table 1.** Eight-week mindfulness curriculum for older adults.

Week	Theme	Course outline	Formal practice	Digital learning materials
Introduction	Orientation	Course introduction, mindfulness breathing, awareness exercises, group building, and instruction on using digital course videos (LINE/YouTube)	Mindfulness breathing	—^a^
1	Mindfulness awareness experience	Mindful breathing, intention meditation, body scan, 5-sense awareness, and mindful stretching	Mindful breathing, body scan, mindful stretching, and yoga	Mindful breathing and body scan
2	Perception and creative response	Mindful breathing, sharing of home practice, body scan, mindful stretching, mindful eating, and 9-point practice	Body scan, mindful stretching, yoga, and mindful eating	Mindful breathing, body scan, mindful stretching (standing), and mindful eating
3	Experiencing the joy and strength of the present moment	Mindful breathing, mindful stretching, sharing of home practice, “awareness triangle” for appreciating good things, mindful eating and drinking	Breathing observation, mindful stretching, and mindful eating and drinking	Mindful breathing, body scan, mindful eating, and mindful stretching (lying down)
4	Habits and perspectives: how interpretations shape experience	Mindful breathing, mindful stretching, sharing of home practice, application of mindfulness in daily life, recognizing thoughts, sharing pleasant events, in-class exchange, and loving-kindness meditation	Mindful breathing, mindful stretching, yoga, and loving-kindness meditation	Mindful breathing, body scan, and loving-kindness meditation
5	Discovering space and possibilities in choice	Mindful breathing, mindful stretching, non-judgmental awareness practice, midterm review and sharing, awareness of unpleasant events and mind-body connection, mindful walking, in-class exchange, and loving-kindness meditation	Mindful breathing, mindful walking, and loving-kindness meditation	Mindful breathing, body scan, mindful walking, and loving-kindness meditation
6	Stress and automatic pilot (STOP)	Mindful breathing, mindful stretching, sharing of home practice and daily application of mindfulness, mindful eating, understanding stress and stressors, body scan, and STOP practice (stop, take a breath, observe, and proceed), and loving-kindness meditation	Mindful breathing, body scan, STOP practice, and loving-kindness meditation	Mindful breathing, body scan, mindful eating, and mindful stretching
7	Integrating mindfulness into daily life	Mindful breathing, mindful stretching, home-practice sharing, mindful eating, mindful listening and communication, in-class exchange, mindful walking and empathy practice, and loving-kindness meditation	Breathing observation, mindful walking, mindful listening, and loving-kindness meditation	Mindful breathing, body scan, mindful eating, mindful stretching, and mindful listening and communication
8	A new life with mindfulness	Mindful breathing, mindful stretching, sharing of home practice, body scan, group sharing, application of mindfulness in daily life, loving-kindness meditation, and graduation ceremony	Mindful breathing, body scan, mindful stretching, yoga, and loving-kindness meditation	Mindful breathing, body scan, mindful eating, and mindful life

a Not applicable.

**Table 2.** Delphi results from rounds 2 and 3.

Session^a^	Round 2		Round 3	
	Median (IQR)	Agreement (%)	Median (IQR)	Agreement (%)
Introduction	4 (3-4)	88.33	4 (3.25-4)	88.75
Session 1	4 (4-4)	92.59	4 (4-4)	94.32
Session 2	4 (4-4)	95.15	4 (4-4)	91.67
Session 3	4 (4-4)	96.67	4 (4-4)	95.31
Session 4	4 (4-4)	96.67	4 (4-4)	97.60
Session 5	4 (4-4)	98.79	4 (4-4)	99.11
Session 6	4 (4-4)	96.11	4 (4-4)	97.60
Session 7	4 (4-4)	97.04	4 (4-4)	98.75
Session 8	4 (4-4)	94.55	4 (4-4)	99.43

a Items were rated on an ordinal scale; the results are presented as median (IQR) and percentage agreement (rated as “suitable” or “very suitable”). Consensus was defined a priori as IQR≤1.0 and agreement ≥80%.

### Phase 2: Pilot Study

#### Recruitment, Retention, and Missing Data

Ten older adults participated in the pilot study. All completed the 8-week mindfulness program, the quantitative assessments at all 4 time points (P0-P3), and the postprogram qualitative interview. There was no attrition, and quantitative analyses were conducted using complete cases (n=10).

#### Participant Characteristics

All 10 participants were community-dwelling older women with an average age of 77.4 (SD 6.2) years. The majority were retired and had attained a junior high school education or below (n=7, 70%), with monthly incomes under NT $20,000 (US $645; n=9, 90%). Most had experienced widowhood (n=7, 70%) and practiced Buddhism while living with either children or spouses (n=7, 70%). They had multiple chronic health conditions, including musculoskeletal disorders (n=5, 50%), hypertension (n=4, 40%), diabetes (n=3, 30%), and cardiovascular diseases (n=3, 30%). All participants maintained complete independence in their activities of daily living (mean 100, SD 0; Table 3).

**Table 3.** Participant characteristics (N=10).

Characteristic and item	Value
Age (y), mean (SD)	77.4 (6.20)
Gender	
Female	10 (100)
BMI, mean (SD)	26.25 (3.82)
ADL^a^, mean (SD)	100 (0)
Education level, n (%)	
Junior high school and below	7 (70)
Senior school and above	3 (30)
Currently employed, n (%)	
Yes	1 (10)
No	9 (90)
Religion, n (%)	
No	1 (10)
Yes	9 (90)
Marital status, n (%)	
Married	2 (20)
Single/widower/divorced	8 (80)
Monthly income, n (%)	
NT $20,000 (US $645) and below (low income)	9 (90)
NT $20,000 (US $645) and above	1 (10)
Chronic conditions, n (%)	
Hypertension	4 (40)
Diabetes	3 (30)
Hyperlipidemia	2 (20)
Cardiovascular disease	3 (30)
Musculoskeletal disorders	5 (50)
Other	2 (20)
Living alone, n (%)	3 (30)
Yes	3 (30)
No	7 (70)

a ADL: activities of daily living.

#### Quantitative Outcomes

Table 4 presents medians and IQRs for all outcomes across the 4 time points (P0-P3). Friedman tests revealed statistically significant changes over time in mindfulness (FFMQ), sleep quality (PSQI), aging perceptions (B-APQ), healthy aging perspectives (HAPQ), and physical function (SPPB). No significant changes were observed in cognitive function (MoCA), physical activity (PASE-C), or HRV indices (SDNN and LF/HF ratio).

Mindfulness levels also increased over time. Median FFMQ scores rose from 139.50 (IQR 102.25-155.75) at baseline to 150.00 (IQR 141.75-156.25) at week 8 and 155.00 (IQR 136-164.25) at follow-up (*χ*²_3_=11.57, *P*=.009). Post hoc comparisons indicated significantly higher mindfulness at follow-up than at baseline (P3>P0), suggesting sustained improvement in mindfulness skills after the intervention.

**Table 4.** Changes in healthy aging outcomes across 4 time points.

Variable^a^	Baseline (P0) median (IQR)	Week 4 (P1) median (IQR)	Week 8 (P2) median (IQR)	Follow-up (P3) median (IQR)	*χ*^2^ (df)	*P* value	Post hoc
HRV^b^- SDNN^c^	33.15 (21.08-83.42)	25.70 (17.46-73.57)	156.84 (36.10-215.47)	28.78 (19.63-120.74)	1.20 (3)	.75	—^d^
HRV-LF/HF ratio^e^	0.93 (0.54-2.02)	2.05 (1.11-4.63)	2.40 (1.21-4.18)	0.88 (0.69-3.99)	6.60 (3)	.09	—
FFMQ^f^	139.50 (102.25-155.75)	135.00 (124.5-164)	150.00 (141.75-156.25)	155.00 (136-164.25)	11.57 (3)	.009	P3>P0
MoCA^g^	29.00 (26.75-30.00)	29.50 (28.50-30.00)	30.00 (28.50-30.00)	30.00 (28.75-30.00)	3.98 (3)	.26	—
PSQI^h^	8.50 (2.75-11.25)	9.00 (2.75-10.25)	3.50 (1.00-6.25)	2.50 (1.00-4.50)	15.03 (3)	.002	P3>P0, P2>P0
B-APQ^i^	3.25 (2.96-4.06)	3.63 (3.03-4.53)	3.58 (3.42-4.22)	4.23 (3.57-4.80)	10.88 (3)	.01	P3>P0
HAPQ^j^	3.14 (3.01-3.22)	3.68 (3.30-4.23)	3.78 (3.52-3.94)	4.13 (4.09-4.69)	20.70 (3)	<.001	P3>P0, P2>P0
PASE-C^k^	105.16 (89.67-150.36)	91.36 (61.25-165.01)	67.02 (57.35-139.94)	118.52 (73.19-127.96)	2.03 (3)	.57	—
SPPB^l^	12.00 (11.50-12.00)	11.50 (11.00-12.00)	12.00 (11.75-12.00)	12.00 (12.00-12.00)	8.37 (3)	.04	—

a PSQI: lower scores indicate better sleep quality. FFMQ, B-APQ, HAPQ, and SPPB: higher scores indicate more favorable outcomes.

b HRV: heart rate variability.

c SDNN: standard deviations of normal-to-normal.

d Not applicable.

e LF/HF ratio: low frequency/high frequency ratio.

f FFMQ: Five Facet Mindfulness Questionnaire.

g MoCA: Montreal Cognitive Assessment.

h PSQI: Pittsburgh Sleep Quality Index.

i B-APQ: Brief Aging Perceptions Questionnaire.

j HAPQ**: **Healthy Aging Perspectives Questionnaire.

k PASE-C: Physical Activity Scale for the Elderly.

l SPPB: Short Physical Performance Battery.

Sleep quality improved markedly: median PSQI scores decreased from 8.50 (IQR 2.75-11.25) at baseline to 3.50 (IQR 1.00-6.25) at week 8 and 2.50 (IQR 1.00-4.50) at follow-up (*χ*²_3_=15.03, *P*=.002). Post hoc comparisons showed significantly lower scores at P2 and P3 than at P0, indicating progressively better sleep.

Perceptions of aging and healthy aging perspectives also became more positive. Median B-APQ scores increased from 3.25 (IQR 2.96-4.06) at baseline to 4.23 (IQR 3.57-4.80) at follow-up (*χ*²_3_=10.88, *P*=.01), with post hoc tests indicating higher scores at P3 than P0. Median HAPQ scores increased from 3.14 (IQR 3.01-3.22) at baseline to 3.78 (IQR 3.52-3.94) at week 8 and 4.13 (IQR 4.09-4.69) at follow-up (*χ*²_3_=20.70, *P*<.001), with significantly higher scores at P2 and P3 compared with P0.

Physical function remained high and showed small but statistically significant variation over time: median SPPB scores were 12.00 (IQR 11.50-12.00) at baseline, dipped slightly to 11.50 (IQR 11.00-12.00) at week 4, and returned to 12.00 (IQR 11.75-12.00) at week 8 and to 12.00 (IQR 12.00-12.00) at week 12 (*χ*²_3_=8.37, *P*=.04).

By contrast, cognitive function (MoCA) remained consistently high (≈29.0) at all time points, and median PASE-C and HRV indices (SDNN and LF/HF ratio) fluctuated without a clear trend. None of these variables changed significantly over time (all *P*>.05).

#### Qualitative Outcomes

Thematic analysis of the postprogram interviews revealed 5 main themes, each with several subthemes, describing the physical and psychological sensations experienced after participating in the digitally supported mindfulness program (Table 5):

- Enhanced physical and psychological well-being: participants reported greater relaxation, better sleep, and improved emotional regulation.
- Transformation of daily routines: mindfulness practices gradually shifted from occasional use to habitual strategies for coping with discomfort and stress in everyday life.
- Memorable practices and preferred activities: simple, accessible practices (eg, deep breathing, bed-based stretching, and mindful eating) were viewed as most useful and sustainable.
- Positive life changes and social connectedness: participants described adopting healthier behaviors, shortening periods of negative emotions, and experiencing reduced loneliness through group interactions.
- Program evaluation and suggestions: the overall satisfaction was high. Digital tools (LINE and YouTube) were perceived as helpful, although some participants with reading difficulties needed family assistance to access online content and requested continued classes.

Taken together, the qualitative findings indicate that participants experienced meaningful benefits from the digitally supported mindfulness program, including improved relaxation, emotional regulation, and social connectedness. Accessible practices centered on breathing and yoga were most consistently adopted into daily life. Although digital tools were widely valued for supporting home practice, a subset of participants reported accessibility challenges, highlighting the need for age-friendly digital solutions.

**Table 5.** Themes, subthemes, and illustrative quotes from qualitative interviews.

Theme and subtheme	Illustrative quote
Enhanced physical and psychological well-being	
Improved relaxation and sleep	*Sometimes when I cannot fall asleep, I just take deep breaths and stretch my legs in bed. Soon, I feel more comfortable and can fall asleep.* (participant C) *Prior to participating in this course, I experienced significant difficulties with sleep; however, following the practice sessions, my sleep quality has shown gradual improvement.* (participant H)
Emotional stabilization and stress relief	*When I am busy and feel upset, I just take a few deep breaths. It really helps me relax, and my mood improves.* (participant A)
Transformation of daily routines	
From passive learning to habitual practice	*At first, I often forgot. But when I saw the YouTube videos in the LINE group, I remembered and did the exercises at home.* (participant F)
From reactive to proactive coping	*When my chest hurt, I first thought about doing deep breathing. I tried hard to breathe in, and after a while, I felt better.* (participant B)
Memorable practices and preferred activities	
Breathing as a core practice	*Deep breathing is the most useful. I can do it anywhere, and it calms me quickly.* (participant H)
Bed-based and stretching exercises	*I like the bed exercises the most. Even when it’s cold, I can do them under a blanket. They really loosen my body.* (participant D) *During my stretching exercises, I have started to focus closely on the sensations in my body with each movement, especially to pinpoint the exact areas where I feel exertion.* (participant E)
Mindful eating and slow chewing	*Now I chew more slowly and carefully. It helps me taste the food and prevents me from choking.”*(participant G)
Positive life changes and social connectedness	
Adoption of healthier behaviors	When I experience a negative mood, I practice mindful walking in the park to help alleviate it. (participant F) *I eat slower, drink more water, and keep moving every day. It’s a real change for me.* (participant E)
Improved emotional regulation	*When bad things happen, I shorten the time I feel sad or angry. I try not to hold onto it for too long.* (participant D)
Reduced loneliness and enhanced social ties	*Coming here and practicing with others makes me feel less lonely. At home, I would think too much.* (participant H)
Program evaluation and suggestions	
High satisfaction and appreciation	*I really enjoy these classes. The teacher’s voice is very soothing.* (participant G)
Role of digital technology	*I don’t read well, so I rarely check the YouTube videos myself. My daughter helps me open them.* (participant I)
Desire for continuity	*I hope you can come again regularly. If we keep practicing together, we won’t forget.* (participant A)

## Discussion

### Principal Findings

In this mixed methods feasibility study, we developed and piloted an 8-week digitally supported mindfulness curriculum integrating MBSR, MBEC, and MSA frameworks for community-dwelling older adults in Taiwan. Assessments at 4 time points revealed significant improvements in mindfulness and sleep quality, along with more positive attitudes toward aging. However, physical function showed only a slight (but significant) improvement, and no significant changes were observed in cognitive function, physical activity, or HRV indices. The quantitative data were supported by the qualitative interview results, which indicated that participants developed more effective relaxation and emotional regulation techniques and became more capable in daily activities and interpersonal relationships. Overall, the findings indicated that the program was successful, with participants providing positive feedback on both culturally adapted content and digital support components.

The program integrated MBSR with MBEC and MSA frameworks while adapting content to the cultural and educational contexts of older adults in Taiwan. Mindfulness interventions offered via structured courses and digital platforms require careful cultural adaptation [35], and research highlights the importance of incorporating cultural values, language, social interaction styles, and accessible technology to enhance participant engagement and overall effectiveness [36-38]. In this study, we used 3 strategies to address reported barriers related to low literacy and abstract mindfulness concepts: simplified language, locally relevant exercises, and embodied practices. Participants described the LINE reminders and YouTube videos as useful digital tools that facilitated between-session practice. However, some participants reported the need for family assistance to access and use the materials due to limited reading ability. These findings highlight both the potential and practical challenges of digital support for older adults and underscore the importance of age-friendly, accessible interface design.

The observed improvement in sleep quality is consistent with participants’ descriptions of using breathing techniques and gentle stretching to manage sleeplessness. By reducing presleep cognitive arousal and facilitating relaxation, these practices likely contributed to the marked decline in PSQI scores. This finding aligns with previous research showing that mindfulness training improves sleep quality by supporting emotional regulation and reducing stress-related hyperarousal symptoms, which can affect older adults [39].

Improvements in aging perceptions and healthy aging perspectives reflect the cognitive reappraisal and acceptance techniques emphasized by both the MBEC and MSA approaches. Mindfulness practices enable individuals to distance themselves from unpleasant thoughts, cultivate self-compassion, and recognize meaningful moments throughout their day. Participants reported experiencing shorter episodes of negative emotions as they learned to manage difficult situations, which align with an adaptive aging orientation. This transition may have significant health implications, as individuals who believe they are aging well tend to preserve their physical abilities and remain active in later life [2,4].

### Factors Contributing to Unchanging Variables

Median SPPB scores remained high throughout and showed a small but statistically significant improvement, whereas physical activity levels assessed by the PASE-C did not change significantly. This outcome is consistent with previous studies demonstrating that mindfulness training produces variable improvements in physical movement quality, balance, and mobility [14,40,41]. The curriculum focused on teaching safety and awareness during light exercise rather than increasing overall exercise duration; consequently, changes in short mindful movement sessions—such as bed-based stretching and mindful walking—are unlikely to be captured by the PASE-C, which primarily measures the frequency of leisure activities and household tasks. Although mindfulness training does not aim to increase activity duration, it can facilitate improvement in movement quality and physical awareness during these exercises.

Despite participants’ numerous positive subjective reports, no statistically significant improvement was observed in cognitive function as measured by MoCA. This finding is likely attributable to the ceiling effect. Participants showed normal cognitive abilities at baseline, as their MoCA scores were already near maximum and concentrated within a narrow range. The MoCA test fails to detect small changes in executive function and attention that mindfulness may produce in cognitively healthy older adults under specific conditions. Future research should include a more diverse group of participants who remain under observation for an extended period while using various cognitive tests to measure the effectiveness of interventions [12].

The HRV indices (SDNN and LF/HF ratio) did not show significant variations. However, HRV can fluctuate widely owing to factors such as the testing environment, breathing patterns, body position, measurement timing, and concurrent medications. Meta-analytic research indicates that mindfulness practices have small and variable effects on HRV, which are influenced by vagal nerve function [42,43]. Future studies should aim to determine when physiological changes occur by employing larger sample sizes, longer observation periods, and standardized resting-state measurement protocols.

### Limitations

Several study limitations should be noted. First, the small sample size (N=10) and single-site recruitment reduce the statistical power and generalizability of the findings. Second, since the majority of older adults at the Long-Term Care Service C Stations are female, all participants were women, restricting the applicability of findings to older men [44]. Third, the PASE-C instrument lacked the sensitivity to detect changes in mindful light-intensity movements, which likely contributed to the null finding in physical activity levels. Finally, although digital tools supported program engagement, several participants with limited literacy required family assistance to access online materials, emphasizing the need to create digital solutions that are both age-friendly and inclusive.

### Links With Prior Evidence, Implications, and Future Directions

Our findings support previous studies showing that mindfulness programs benefit older adults’ sleep quality and mental health [12] by demonstrating that similar gains are achievable within a digitally supported, community-based format that also enhances aging-related perceptions and functional outcomes. This hybrid learning model—combining weekly in-person sessions with LINE reminders and instructor-recorded videos—offers a potentially cost-effective approach to sustaining home practice and maintaining engagement in real-world settings, addressing common challenges to program adherence [13].

Based on this pilot study, findings should be interpreted as preliminary evidence of feasibility and perceived usefulness rather than as a basis for immediate large-scale implementation. The program structure may nonetheless inform future evaluations at Long-Term Care Service C Stations and other community-based aging services, serving as a potential model for incorporating brief mindfulness practices—such as mindful breathing, chair-based stretching, and brief body scans—into regular group activities. A train-the-trainer approach supported by a structured manual (including session scripts, safety cues, and culturally relevant examples) may facilitate standardized delivery across sites. Importantly, suggestions regarding age-friendly digital interfaces (eg, larger text, simplified navigation, structured screens, and short audio/video materials delivered via familiar platforms) were derived from participants’ reports of accessibility barriers (eg, limited reading ability and reliance on family assistance) rather than from formal usability testing; therefore, these design implications should be validated in future work using established usability evaluation methods.

Future research should assess program effectiveness and scalability using adequately powered randomized controlled trials with longer follow-up, objective activity monitoring, more sensitive cognitive measures, standardized protocols, and hybrid effectiveness-implementation designs that assess both outcomes and adoption metrics (eg, reach, fidelity, and cost). Formal usability evaluation (eg, task-based testing and validated usability measures) and objective engagement metrics should also be incorporated to optimize the digital support components.

### Conclusion

This study makes a dual contribution: it demonstrates expert consensus on the content validity of a culturally adapted, theory-grounded mindfulness curriculum for community-dwelling older adults in Taiwan, and it provides preliminary evidence of the program’s feasibility, acceptability, and beneficial effects across psychological, sleep-related, and aging perception domains. The hybrid delivery format was generally well-received and appeared to support home practice. Importantly, this work extends existing research by showing that mindfulness benefits are not only confined to sleep and mental health but also extend to how older adults perceive and engage with aging. Additional trials are needed to confirm these preliminary effects, refine assessment methods, and establish conditions for scalability.

## Supplementary material

10.2196/84161 Multimedia Appendix 1 Expert characteristics.

10.2196/84161 Multimedia Appendix 2 Semistructured interview guide.

10.2196/84161 Multimedia Appendix 3 Research measurements.

## Abbreviations

B-APQ: Brief Aging Perceptions Questionnaire
FFMQ: Five Facet Mindfulness Questionnaire
HAPQ: Healthy Aging Perspectives Questionnaire
HF: high frequency
HRV: heart rate variability
LF: low frequency
MBEC: mindfulness-based elder care
MBI: mindfulness-based intervention
MBSR: mindfulness-based stress reduction
MoCA: Montreal Cognitive Assessment
MSA: mindful sustainable aging
PASE-C: Physical Activity Scale for the Elderly
PSQI: Pittsburgh Sleep Quality Index
SDNN: standard deviation of normal-to-normal
SPPB: Short Physical Performance Battery
WHO: World Health Organization
