# Supplementary material for: Using a Digital-Based Mindfulness Curriculum to Enhance Healthy Aging Outcomes in Community-Dwelling Older Adults in Taiwan: Mixed Methods Feasibility Study
Source: JMIR Hum Factors. 2026 May 13;13:e84161. doi: 10.2196/84161 (PMC13170997; doi:10.2196/84161)
Supplement: Multimedia Appendix 1 [file humanfactors-v13-e84161-s001.docx]

**Multimedia Appendix**

**Table S1. Expert characteristics.**

| Expert | Gender | Education | Work experience | Years of experience |
| --- | --- | --- | --- | --- |
| A | Male | Master’s | Clinical psychologist and MBSR instructor | 12 |
| B | Male | Master’s | Counseling psychologist and MBSR instructor | 6 |
| C | Female | Master’s | Nurse practitioner and MBSR instructor | 9 |
| D | Male | Bachelor of Medicine | Medical education professor, brain science expert, and MBSR instructor | 9 |
| E | Male | Master’s | MBSR and Mindfulness-Based Cognitive Therapy (MBCT) instructor | 9 |
| F | Male | PhD | Social education and MBSR instructor | 7 |
| G | Male | PhD | Clinical psychologist and brain behavior expert | 5 |
| H | Female | Master’s | Chinese medicine expert and MBSR instructor | 6 |
| I | Female | Master’s | MBSR instructor | 6 |
| J | Female | PhD | Nurse, nursing professor, and geriatric care expert | 15 |
| K | Female | PhD | Nurse, nursing professor, and geriatric care expert | 15 |
| L | Female | PhD | Nurse, nursing professor, and geriatric care expert | 20 |
| M | Female | Master’s | Case manager and MBSR instructor | 6 |
| N | Female | Master’s | Nurse, psychiatric nurse, and MBSR instructor | 25 |
| O | Male | Bachelor of Science | Occupational therapist, long-term-care expert, and MBSR instructor | 7 |
| P | Male | Master’s | Clinical psychologist and MBSR instructor | 13 |
