# Supplementary material for: Using a Digital-Based Mindfulness Curriculum to Enhance Healthy Aging Outcomes in Community-Dwelling Older Adults in Taiwan: Mixed Methods Feasibility Study
Source: JMIR Hum Factors. 2026 May 13;13:e84161. doi: 10.2196/84161 (PMC13170997; doi:10.2196/84161)
Supplement: Multimedia Appendix 3 [file humanfactors-v13-e84161-s003.docx]

**Multimedia Appendix**

**Research measurements**

**Mindfulness Awareness**

Baer et al. used a Five Facet Mindfulness Questionnaire (FFMQ) for measurement purposes [7]. This tool consists of 39 items that assess five aspects of mindfulness, such as observing, describing, acting with awareness, non-judging, and non-reactivity, on a 5-point Likert scale. The Taiwanese FFMQ [23] demonstrates good psychometric properties, with a Cronbach’s alpha 0.74 and 0.88, an overall alpha of 0.88, and a test-retest reliability coefficient of 0.95. They have also become popular among seniors.

**Heart Rate Variability (HRV)**

Autonomic function assessment used the BEATINFO heart rate sensor (Singular Wings Medical) which has received the TFDA medical device license. It can continuously measure SDNN and the LF/HF ratio. The BEATINFO Health App provided older people’s data to researchers for the analysis of sympathetic–parasympathetic balance. The SDNN value was the standard deviation of the heartbeat interval, and the larger the standard deviation, the greater the variability between heart rates. The normal LF/HF ratio was 1.2 – 2.8.

**Cognitive Function**

The Montreal Cognitive Assessment (MoCA) [24] was used to evaluate cognitive function. Test scores range from 0 to 30, with normal cognition defined as scores of 26 and above in the Taiwanese version [25]. This study demonstrated that the test proved useful for identifying mild cognitive impairment (sensitivity=92%, specificity=78%).

**Sleep Quality**

The Pittsburgh Sleep Quality Index (PSQI) was used to evaluate sleep quality [26]. The 19-item instrument generates a global score ranging from 0 to 21; with scores >5 indicating poor sleep quality. Tsai et al. found that the internal consistency of the Chinese version was satisfactory (Cronbach’s α=0.83) [27].

**Perceptions of Aging**

Brief Aging Perceptions Questionnaire (B-APQ) was used to measure perceptions of aging [28, 29]. In the Taiwanese version, for a set of 18 items, the content validity index was 0.9–1.0, and with adequate reliability (Cronbach’s α=0.86–0.91; overall α=0.87) [30].

**Healthy Aging Perspectives**

The Healthy Aging Perspectives Questionnaire (HAPQ) [31], a 21-item measure developed in Taiwan, and based on the WHO’s conceptual model of healthy aging, assesses how older adults, especially with chronic illnesses, understand and engage with the concept of healthy aging, making it highly relevant to our target population and research objectives. Its four-factor structure received support from psychometric testing which showed acceptable reliability (Cronbach’s α=0.71–0.89; overall α=0.80).

**Physical Activity and Function**

Physical activity was assessed with the Chinese version of the Physical Activity Scale for the Elderly (PASE-C), which had good reliability (ICC=0.85) and concurrent validity with grip strength and depression levels [32]. The Short Physical Performance Battery (SPPB) measured functional performance through a balance test, 4-m walk, and chair stand tests; it produced scores from 0 to 12 points where less than 10 points indicated mobility impairment [33].
